# The impact of VaKE-driven online discussions on critical thinking styles among Chinese undergraduates

**DOI:** 10.3389/fpsyg.2025.1494055

**Published:** 2025-05-01

**Authors:** Xiaoshu Xu, Yujie Su, Huanhuan Zhang, Vivian Ngan-Lin Lei, Xiaofang Ye

**Affiliations:** ^1^School of Foreign Studies, Wenzhou University, Wenzhou, China; ^2^Faculty of Applied Sciences, Macao Polytechnic University, Macau, Macao SAR, China

**Keywords:** values and knowledge education (VaKE), critical thinking styles, online discussion forums, undergraduates, China

## Abstract

This study examines the impact of VaKE-guided online discussion forums on the critical thinking (CT) styles of 100 undergraduate students in Southeast China, focusing on engagement and information-seeking tendencies. Unlike previous research on VaKE’s impact on CT skills, this study investigates its effect on CT styles. A mixed-methods approach was used, combining the 24-item UFCTI and student reflective diaries. Results showed that the experimental group, engaged in VaKE-guided forums, exhibited improved engagement and information-seeking behaviors, while the control group, using traditional methods, saw a decline. Qualitative data further supported these findings, with students reporting enhanced understanding of social issues and increased cognitive flexibility. Challenges, including insufficient teacher-student interaction, were noted, emphasizing the need for careful facilitation. These findings contribute to the literature on ethical reasoning and CT styles, providing insights for educators seeking to enhance cognitive and moral development in higher education.

## 1 Introduction

Critical thinking (CT) style refers to an individual’s preferred approach to processing information and engaging in critical analysis. These styles can range from actively seeking information to participating in interactive, discussion-based activities. Understanding CT styles is crucial for educators, as it allows them to tailor teaching methods to align with students’ learning preferences, thereby enhancing engagement and fostering the development of CT skills (Saprudin et al., 2019). Research has shown a significant relationship between thinking styles and critical thinking abilities, with certain styles, such as executive thinking, positively correlating with higher critical thinking scores (Abdi, 2012). This highlights the importance of understanding and cultivating diverse thinking styles to enhance critical thinking capabilities.

A review of the literature indicates the research on critical thinking styles can be categorized into three main areas: identifying different styles, developing assessment tools, and exploring strategies to modify these styles. In terms of CT style identification, Lamm and Irani’s (2011) University of Florida Critical Thinking Inventory (UFCTI) makes an accurate assessment, distinguishing between “Seekers,” who actively pursue infomation and prefer to take a meticulously researched, approach to their decision-making, and “Engagers,” who thrive in interactive settings and benefit from open discussions within group settings (Gay et al., 2015; Lamm and Irani, 2011). Beyond these, other research has expanded the classification of thinking styles into five types: superior creative thinking (ScreT), creative thinking (CreT), balanced thinking (BT), critical thinking (CriT), and superior critical thinking (SCriT), each influencing problem-solving and decision-making differently (Saprudin et al., 2019). Saprudin et al. believed in the definition of CT involving disposition and abilities. CT refers to the ability of elementary clarification, basic support, inference, advanced clarification, and strategy and tactic. Additionally, they adopted the idea of Cotrrell’s CT skills: identifying, evaluating, weighing up opposing arguments and evidence fairly, interpreting, judging, drawing conclusions, and expressing points of view. In order to design a model of gamification on physics learning content to develop CT skills, they further put forward the idea of mapping the pre-service physics teachers’ CT skills and the thinking style. However, Saprudin et al. did not give evidence to support for the mapping.

To assess CT styles, several tools have been developed. The UFCTI, developed by Lamm and Irani (2011), is particularly valuable in educational settings as it helps tailor teaching strategies to better align with students’ CT styles (Gay et al., 2015). Complementing the UFCTI, the Yanpiaw Creative-Critical Styles Test, designed to assess the thinking styles of pre-service physics teachers, and the California Critical Thinking Skills Test (CCTST), which evaluates core critical thinking skills, provide further insights into individuals’ cognitive tendencies (Saprudin et al., 2019; Abdi, 2012). These assessment tools are integral to educational research and practice, facilitating a deeper understanding of students’ cognitive styles and preferences.

Modifying CT styles is another area of focus, with educators employing various strategies to enhance critical thinking. Techniques such as reflective audiotaped journals, computer simulations, and content-based approaches have been shown to promote critical and reflective thinking (Dantas-Whitney, 2002; Yeh and Chen, 2003; Liaw, 2007). Tailoring educational strategies based on assessments like the UFCTI allows for more effective engagement with students, with activities designed to suit the specific CT styles identified (Gay et al., 2015; Lamm and Irani, 2011). Active learning environments, such as blended learning settings, further support CT style development by encouraging collaboration and reflection (Graham, 2005; Howard et al., 2006). Additionally, student support services can use CT style assessments to offer personalized guidance, aligning study techniques and resources with students’ cognitive strengths (O'Sullivan and Guo, 2010). Program evaluations using CT style assessments help educators adjust strategies to enhance critical thinking outcomes, though the effectiveness of interventions also depends on factors like teaching methods, student motivation, and classroom dynamics (Lun et al., 2010).

Despite extensive research on CT styles, most studies have primarily focused on identifying these styles in various contexts rather than exploring effective methods for modifying them. While tools such as the UFCTI have been validated across different cultural settings, there is a notable lack of research into strategies that can actively alter CT styles. Moreover, existing research has predominantly concentrated on specific fields like agriculture and natural resources, underscoring the need for studies that span a wider range of disciplines and professional backgrounds. Disciplinary knowledge is an intrinsical prerequisite for interdisplinarity, and only when the original disciplines are integrated into broader, more comprehensive framework can interdisciplinary recognized the solutions to particular problems (Mazzocchi, 2019).

The Values and Knowledge Education (VaKE) method is an instructional strategy that integrates scientific knowledge acquisition with ethical reasoning, aligning closely with the development of CT styles. VaKE presents students with moral dilemmas that require deep engagement in critical thinking, argumentation, and reasoning, aligning with the CT styles of engagement and information-seeking (Pnevmatikos et al., 2016). Through VaKE, students learn to critically assess their values, distinguish between beliefs and evidence, and reflect on the ethical implications of their decisions. This process not only enhances critical thinking development (CTD) and critical thinking skills (CTS) but also encourages a shift from intuitive to analytical, rule-based reasoning (Evans, 2008).

While substantial research has been conducted on critical thinking in higher education, most studies have focused on traditional classroom settings or general online learning environments (Hu, 2024; Kiran et al., 2023; Park and Choi, 2014), explored the traditional teaching and learning and concluded the defects, passive thinking in particular, and none had specifically examining the role of forum discussions in enhancing critical thinking. The potential of online forums to facilitate critical engagement, asynchronous debate, and deep reflection remains largely underexplored, highlighting a significant gap in the literature. Furthermore, there is a limited understanding of how online discussion forums, particularly those guided by the VaKE method, can be utilized to modify critical thinking styles among undergraduates.

This study aims to fill the gap by exploring the impact of VaKE-driven online discussions on modifying critical thinking (CT) styles among undergraduates in China. The research seeks to inform and enhance educational practices, particularly in digital learning environments, by examining how online forum discussions shaped by the VaKE method influence critical thinking.

The study will investigate the shift in undergraduates’ CT styles through pre-and post-intervention assessments and analyze the insights from reflective diaries. The goal is to provide empirical evidence on how online discussion forums, integrated with the VaKE approach, can affect critical thinking styles in higher education. Specifically, the research will address the following questions:

(1) How do critical thinking styles influence participation in VaKE-guided online discussions among Chinese undergraduates?
(2) How do online forums structured around the VaKE approach impact the development of critical thinking skills in Chinese students?
(3) What role does instructor facilitation play in enhancing critical thinking and engagement in VaKE-driven online discussions?

## 2 Literature review

### 2.1 Theoretical background of VaKE framework

VaKE is an innovative educational approach that seamlessly integrates moral and cognitive development within a constructivist framework. Rooted in the principles of constructivism, VaKE posits that learners actively construct their understanding of the world through experience and social interaction. It leverages this foundation to create inquiry-based learning environments where learners identify their knowledge gaps and actively seek information to address them (Baviskar et al., 2009).

The VaKE method incorporates Kohlberg’s theory of moral development, which outlines stages of moral reasoning that individuals progress through. Kohlberg’s Psychology of Moral Development addresses philosophical, psychological, and educational dimensions, with the most significant transition in the 1970s, when Kohlberg began distinguishing between the structure and content of moral judgments. He argued that while the formal and structural aspects of morality are universal, the content of moral judgment can vary across cultures (Naito, 2013). This distinction between structure and content is essential for understanding how VaKE adapts Kohlberg’s framework to focus on the universal elements of moral development while being flexible to cultural contexts.

Though Kohlberg’s hierarchical stages have been critiqued, particularly the assumption that higher stages of moral reasoning (e.g., deontological reasoning at stages 5 and 6) are superior to lower stages (e.g., utilitarian reasoning at stage 2), VaKE engages students in moral dilemma discussions that foster reflection and critical thinking. These discussions aim to create cognitive disequilibrium, prompting learners to reflect on their values and beliefs, refine their moral arguments, and engage with multiple perspectives (Kohlberg, 1984; Blatt and Kohlberg, 1975). While Kohlberg’s hierarchy provides a foundational structure, VaKE encourages a more dynamic, context-driven approach to moral development, acknowledging that moral reasoning evolves through reflection and social interaction.

A key component of VaKE is its focus on social problem-solving, highlighting the collaborative nature of learning. Through group discussions, learners collaboratively address problems, share diverse perspectives, and provide constructive feedback to assess the viability of potential solutions (Bailey and Im-Bolter, 2020).

Additionally, VaKE places a strong emphasis on developing critical thinking skills, essential in today’s information-rich environment. It encourages learners to critically evaluate the reliability and validity of the information they encounter and engage in discourse that challenges existing assumptions (Fan et al., 2024).

Moreover, VaKE aligns with the principles of collaborative learning, recognizing that the exchange of ideas and mutual support among learners significantly enhances their understanding of the subject matter. This collaborative approach is central to VaKE, facilitating a deeper and more comprehensive grasp of the topics studied (Vygotsky, 1978).

In summary, VaKE is a holistic educational approach that not only develops learners’ cognitive and moral capacities but also equips them to be active, critical, and responsible participants in a diverse and interconnected world.

### 2.2 VaKE approach in education

VaKE is a multifaceted constructivist approach that integrates moral reasoning with the active construction of knowledge. VaKE begins by presenting learners with moral dilemmas, prompting them to engage in reflection, discussion, and inquiry-based information searches to deepen their understanding and substantiate their moral arguments (Weinberger et al., 2009; Pnevmatikos et al., 2016; Pnevmatikos and Christodoulou, 2018). This process stimulates critical thinking and exposes learners to the complexities of ethical decision-making.

The iterative nature of VaKE, which involves synthesizing new information, re-evaluating positions, and adapting viewpoints based on collective discussion, is central to developing critical thinking skills and a comprehensive understanding of both subject matter and ethical dimensions (Weinberger, 2006). Teachers act as facilitators in this process, guiding and supporting learners while fostering an open and respectful learning environment where diverse viewpoints are welcomed (Nussbaumer, 2022).

VaKE’s adaptability makes it a versatile tool suitable for various educational levels and subjects. It can be integrated into existing curricula to enhance critical thinking, moral reasoning, and knowledge construction, offering a strategic educational enhancement that enriches traditional teaching methods by embedding moral and ethical considerations (Weinberger et al., 2009). The approach promotes student-centered learning, encouraging active participation, questioning, and engagement with relevant societal dilemmas, thereby developing essential skills such as argumentation, reflection, and decision-making (Weinberger, 2006; Nussbaumer, 2022).

Research has demonstrated VaKE’s effectiveness in improving learning outcomes, including knowledge acquisition and application across various subjects (Weinberger and Nussbaumer, 2019). The integration of VaKE into curricula also highlights the need for professional development to ensure its effective implementation (Nussbaumer, 2022). Additionally, assessment within the VaKE framework requires the development of tools that capture the depth of students’ understanding and their ability to apply values and knowledge in different contexts (Patry et al., 2013). Overall, VaKE is positioned as a powerful instructional strategy for fostering critical thinking, moral reasoning, and knowledge construction in contemporary education.

### 2.3 Impact of VaKE on critical thinking

The VaKE method plays a pivotal role in shaping Critical Thinking (CT) styles by integrating ethical reasoning with knowledge acquisition. While it has been well-documented that VaKE enhances Critical Thinking Skills (CTS) such as analysis and evaluation, it also significantly influences critical thinking dispositions or styles, such as open-mindedness and cognitive maturity. This method encourages students to engage in reflective discourse, argumentation, and informed decision-making, thereby supporting the holistic development of critical thinking, particularly within higher education contexts (Pnevmatikos et al., 2016; Patry, 2014, 2016; Evans, 2008; Barnett, 1997).

The relationship between CT skills and CT styles is essential for understanding how educational interventions like VaKE can modify thinking patterns. Sternberg and Waggner (1992) proposed thinking styles inventory, having 24 items that measured three types of students’ thinking styles including the legislative, executive, and judicial styles. Research indicates that specific thinking styles, such as judicial or legislative styles, correlate positively with critical thinking abilities (Abdi, 2012). Thus, enhancing CT skills through methods like VaKE may concurrently influence students’ preferred CT styles, promoting a more balanced and effective approach to problem-solving and ethical reasoning.

Moreover, VaKE’s emphasis on critical reflection—a core component of transformative learning—aligns with the development of sophisticated CT styles. According to Mezirow’s transformative learning theory, critical reflection, autonomy, and the ability to reframe one’s perspectives are essential for deep learning and personal growth (Mezirow, 1991, 2000). VaKE challenges learners to critically examine and potentially revise their beliefs and values, thereby deepening both CT skills and CT styles, fostering analytical reasoning and ethical consideration (Mezirow, 1996). This structured, self-directed learning process encourages a habit of critical reflection, promoting self-awareness and metacognitive skills—traits that are closely associated with mature and effective CT styles.

To fully grasp the impact of VaKE on CT styles, it is crucial to examine how these theoretical benefits manifest in specific educational contexts. For instance, the application of VaKE in pre-service teacher education and professional development has been shown to foster interdisciplinary understanding and critical reflection on personal values in teaching, which in turn, influences the critical thinking styles of these future educators (Keast and Marangio, 2015). This highlights VaKE’s potential not just in enhancing CT skills but also in cultivating more adaptive and context-sensitive CT styles.

Furthermore, evidence from various educational settings supports VaKE’s effectiveness in nurturing deep learning and equipping learners with robust CT skills, which are closely linked to certain CT styles. These implementations demonstrate that VaKE promotes both critical thinking and transformative learning, thereby enabling students to navigate and contribute positively to a complex global society (Pnevmatikos and Patry, 2014; Mezirow, 2003).

In summary, while previous research has primarily focused on VaKE’s impact on developing Critical Thinking (CT) skills, this study will explore its influence on CT styles. Specifically, it will examine how VaKE shapes students’ cognitive tendencies, such as engagement and information-seeking, providing new insights into the method’s potential to foster adaptive and flexible thinking in complex, real-world contexts.

### 2.4 Critical thinking styles

**Figure 1:**
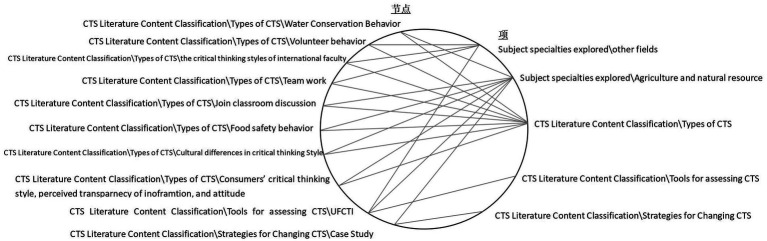
Content categorization of relevant literature and division of research fields.

Extensive research has been conducted on critical thinking styles, and based on a review of the literature, relevant studies can be categorized into three main areas (as illustrated in Figure 1, specifically the third to fifth categories on the right side): examining the types of critical thinking styles among participants, developing and testing tools for assessing critical thinking styles, and exploring strategies to modify these styles.

Firstly, the third category highlights that the majority of studies focus on identifying and examining the types of critical thinking styles among participants. For instance, Lu et al. (2021) explored the differences in critical thinking styles between Chinese and American students, attributing these differences to their diverse cultural backgrounds. Other researchers, such as Barrick and DiBenedetto (2019), have investigated the critical thinking styles of university teachers, emphasizing the significance of educators teaching critical thinking based on their own styles. Additionally, some studies have noted variations in how learners with different critical thinking styles participate in course discussions (Lu et al., 2019). Similarly, research by Owens and Lamm (2016) and Leal et al. (2017) has shown that individuals with varying critical thinking styles exhibit different behaviors in routine activities, such as protecting water resources and ensuring food safety, which require practical decision-making.

Critical thinking styles are often context-dependent, with students demonstrating different approaches depending on the task or discipline at hand (Facione, 2011; Kuhn, 2015). This suggests that students may exhibit multiple CT styles depending on the context, which is an important consideration when assessing their overall critical thinking capabilities.

The fourth category pertains to the development of tools for assessing critical thinking styles and evaluating their effectiveness across different cultural contexts (Owens and Lamm, 2016; Abu-Baker et al., 2021). The final category indicates that only a limited number of studies have explored strategies for changing critical thinking styles. For example, Akins et al. (2019) demonstrated that integrating case studies into agricultural and natural resources courses could enhance students’ tendencies toward seeking and engaging in critical thinking, ultimately improving their critical thinking skills. However, most existing research primarily focuses on identifying participants’ critical thinking styles, with little empirical investigation into teaching strategies that can effectively induce changes in these styles. This gap presents an opportunity for further exploration of strategies to modify learners’ critical thinking styles.

In addition to the previously mentioned categories, the top two categories on the right side of the circular diagram highlight the specific fields studied in existing literature. Most studies focus on the fields of agriculture and natural resources, with limited exploration of participants from other professional or interdisciplinary backgrounds. Given the context-dependent nature of CT styles, it is essential to investigate the critical thinking styles of participants from diverse professional or interdisciplinary contexts, as this may reveal important variations in how critical thinking is applied across disciplines (Facione, 2011). Exploring these areas can provide valuable insights and enhance our understanding of how critical thinking styles vary across different fields.

In conclusion, a review of the existing literature reveals a clear need to explore the types of critical thinking styles among learners across various disciplines and to investigate strategies for effectively modifying these styles.

### 2.5 The University of Florida Critical Thinking Inventory (UFCTI)

The University of Florida Critical Thinking Inventory (UFCTI) was developed in response to a gap identified in the measurement of critical thinking (CT) dispositions by Rudd et al. (2000). Their research suggested that existing tools, such as the California Critical Thinking Disposition Inventory (CCTDI), did not fully capture the constructs of CT as outlined by Facione et al. (1995). This recognition led to the creation of the UF/EMI, which, after a decade of rigorous testing and refinement, evolved into the UFCTI. The UFCTI is grounded in the theoretical understanding that CT is not solely a set of skills but also a cognitive style, reflecting how individuals approach problem-solving and decision-making. The instrument measures CT styles on a continuum between seeking information and engagement, aligning with an individual’s cognitive preferences.

Lu et al. (2019) validated the University of Florida Critical Thinking Inventory (UFCTI) for Chinese undergraduate agricultural students, ensuring cultural and linguistic accuracy. Confirmatory Factor Analysis (CFA) confirmed its structure, with Cronbach’s alpha indicating reliability. Construct validity was supported by metrics like composite reliability (CR) and model fit indices. Despite a limited sample, the study established the UFCTI’s effectiveness in measuring critical thinking styles in the Chinese context, highlighting its potential to improve teaching strategies.

The UFCTI has been widely used to evaluate the construct validity and reliability of critical thinking (CT) across diverse cultural contexts. For instance, Peng Lu and colleagues translated the UFCTI into Chinese and confirmed its reliability and validity among Chinese undergraduate agricultural students (Lu et al., 2019). Such validation studies have demonstrated the UFCTI’s adaptability, making it a crucial tool in various educational settings.

In curriculum development, insights from the UFCTI have enabled educators to design curricula that cater to different CT styles. This approach has allowed for the integration of discussion-based activities for students inclined toward engagement and research-based tasks for those favoring information-seeking, thus creating a more personalized learning experience (Lamm and Irani, 2011). Additionally, faculty members have leveraged UFCTI results to adapt their teaching methods, opting for interactive lectures and group work when a predominant engagement style is identified among students (Gorham et al., 2014).

The UFCTI has also played a role in student support services, where academic advisors use the inventory to offer personalized guidance. This helps in recommending study techniques, resources, and career paths that align with students’ cognitive strengths (O'Sullivan and Guo, 2010). Moreover, the UFCTI has enriched the assessment of learning outcomes, enabling institutions to track the progression of students’ CT skills and evaluate the effectiveness of teaching strategies (Rudd et al., 2000).

In interdisciplinary studies, the UFCTI has identified differences in CT approaches among students from various academic backgrounds, aiding in the design of courses that bridge cognitive disparities and foster cohesive learning experiences (Zhang and Sternberg, 2001). In the context of international education, the UFCTI has been instrumental in assessing CT styles across cultures, promoting an inclusive learning environment that values diverse thinking perspectives (Yeh and Chen, 2003).

Furthermore, honors programs have utilized the UFCTI in their selection processes to identify students with strong CT abilities or to provide focused support for skill development (Lu et al., 2019). The UFCTI has also streamlined program evaluations by comparing CT style profiles at different stages to measure the impact of educational interventions (Lun et al., 2010).

This study uses the University of Florida Critical Thinking Inventory (UFCTI) to investigate the impact of VaKE method-guided online discussion forums on undergraduates’ critical thinking styles.

### 2.6 Online discussion forum

Although the existing empirical research on changing learners’ strategies for critical thinking style is scarce, some studies have proposed ideas in their recommendations section about altering critical thinking styles. These included classroom discussions (Lu et al., 2019), group discussions, and debates (Lu et al., 2021). However, considering the current classroom environment in China on one side, the types of two-way interactive communication mentioned are precisely what Chinese students lack, as Chinese classrooms are generally quiet (Paton, 2005). Classroom debates may be perceived as harmful in the Chinese classroom setting (Tan, 2017). Nevertheless, some studies have urged Chinese educational institutions to change the current education system to better prepare Chinese learners for an international academic environment (Lu et al., 2021). From the perspective of critical thinking styles, Chinese students typically exhibit a critical thinking style that seeks information, participating less in interactive discussions (Nisbett, 2010). However, as the founders of critical thinking styles, Lamm and Irani (2011) pointed out, an ideal critical thinking style should possess qualities aligned with both preferences and select specific critical thinking preferences based on existing information in a particular context. Therefore, it still merits an attempt to use discussion teaching strategies to change the critical thinking styles of Chinese learners.

However, research indicated that traditional classroom discussions, due to time constraints and class size limitations, resulted in limited student participation and contributions to classroom thinking (Cheong and Cheung, 2008). This led to shallow outcomes in class discussions, and the impact on students’ critical thinking was minimal (Cheong and Cheung, 2008). In contrast, Cheong and Cheung (2008) proposed an alternative to traditional classroom discussions, namely online forum discussions. This approach can overcome time constraints, allowing learners to participate flexibly at any time and from any location. It also addressed the issue of superficial discussions. The asynchronous communication in online discussion forums gave learners more time to reflect on what others were saying and how they wanted to respond to these issues. However, there is rare research in the existing literatures on using online discussion forums to change critical thinking styles. Therefore, conducting this type of research is essential to fill the gap in the research field.

While the online discussion forums format offered learners ample opportunities for reflection, Mac Knight (2000) emphasized that, due to questioning being foundational for the development of critical thinking, it was more crucial for learners to pose thoughtful questions and acquire questioning skills during the online discussion process. However, given that learners’ cognitive levels and existing experiences were still in the developmental stage, guidance from educators became necessary, aligning with the conclusion emphasized by Barrick and DiBenedetto (2019) regarding the crucial role of educators in fostering learners’ critical thinking. Mac Knight (2000) highlighted the role of teachers in the online discussion forum environment as being able to pose questions that promote the development of learners’ critical thinking, assisting them in cultivating and applying critical thinking skills. Ingeniously, the VaKE method, underpinned by the Paul-Elder Critical Thinking Framework, is grounded in the Socratic questioning characteristic, providing educators engaged in the discussion, evaluation, and teaching of critical thinking with a set of universal guidelines for questioning. This framework enables educators to pose more profound questions that foster the development of students’ critical thinking in the online forum discussion environment.

Additionally, the VaKE Model comprises 16 steps, encompassing both discussion and information-seeking sections. This alignment resonates with the two styles of critical thinking: the engaging critical thinking style and the information-seeking critical thinking style. However, research on using the VaKE-guided online discussion forum as a means to change teaching techniques related to critical thinking styles remains largely unexplored. Therefore, this study aims to investigate the VaKE-guided online discussion forum as a teaching strategy, and then explore the changes and effects it brings to Chinese learners’ critical thinking style.

## 3 Method

The study utilized a mixed-methods approach to assess the impact of VaKE-guided online forums on undergraduate students’ critical thinking. Quantitatively, the University of Florida Critical Thinking Inventory (UFCTI) measured changes in students’ critical thinking styles before and after the intervention. Qualitatively, reflective diaries captured students’ experiences and perceptions, providing deeper insights into how the forums influenced their critical thinking. This combination of methods offered a comprehensive evaluation of the VaKE approach’s effectiveness.

### 3.1 Sample and sampling

This study employed a convenience sampling method, selecting participants based on their availability within a specific academic environment. The sample consisted of 100 first-year undergraduates from a comprehensive university in East China who volunteered to participate. These participants were drawn from parallel English classes, ensuring comparable academic backgrounds, as evidenced by their closely aligned college entrance English scores.

The students were divided into two groups: a control group, which independently completed essay-writing tasks, and an experimental group, which participated in teacher-facilitated discussion forums using the VaKE method. To control for potential differences in student disciplines, the study ensured a balanced distribution of students across fields of study. The sample was predominantly composed of engineering majors, with a small number of art students in the control group and science students evenly distributed across both groups.

The sample was also predominantly male, with a higher representation of males in both the experimental and control groups. Most participants were between 18 and 20 years old, with a smaller proportion of students aged under 18 or between 21 and 24. To minimize the impact of gender and discipline on the results, these variables were considered in the analysis, and any variability was accounted for when comparing the two groups.

The study adhered to strict ethical standards. The research design, methodology, and ethical considerations were approved by the university’s Institutional Review Board (IRB) (No.20240203). Participants were fully informed about the study’s objectives, procedures, and potential impacts, and provided written consent. Anonymity and confidentiality were rigorously maintained, and participation was entirely voluntary, with the option to withdraw at any time without consequences. All data were securely stored according to institutional guidelines and systematically disposed of upon the study’s completion.

### 3.2 Instruments

The UFCTI served as the primary tool for assessing students’ critical thinking styles before and after their participation in the VaKE-guided forums. This inventory evaluated various dimensions of critical thinking, including analysis, evaluation, and inference, providing a comprehensive understanding of students’ cognitive approaches. Although the UFCTI was originally developed in a Western context, it has been adapted and validated for use with Chinese students, as evidenced by Lu et al. (2019), who validated it for Chinese agricultural students. This validation provided a foundation for its application in this study, where it was used to assess critical thinking styles among Chinese undergraduates. The multiple-choice questions use a Likert scale (1–5), with higher scores indicating stronger critical thinking abilities. The open-ended questions provide qualitative insights into students’ reasoning but are not scored.

To ensure cultural and linguistic accuracy, the Chinese version of the UFCTI was used. The translation process involved two expert translators, and to ensure clarity and comprehension, the instrument was piloted with five students. These students provided feedback on clarity, understanding, and usability, which was incorporated into the final version of the instrument. While the UFCTI’s focus on reflective and practical thinking aligned with the recent educational reforms in China, which emphasized active and student-centered learning, we acknowledged that the instrument may require further adaptation in future studies to better capture the cultural nuances of Chinese students’ thinking styles.

In addition to the UFCTI, reflective diaries were employed exclusively for the experimental group at the end of the experiment. These diaries contained two key questions: “What benefits did you gain from the forum, and what improvements did you suggest?” and “What is cyberbullying? What measures did you think should be taken to address cyberbullying?” These questions were designed to prompt participants to reflect on their experiences in the VaKE-guided forums, providing valuable insights into their perceptions of the forum’s effectiveness, their understanding of cyberbullying, and suggestions for improvement. This qualitative data complemented the quantitative findings from the UFCTI, offering a more comprehensive understanding of the intervention’s impact on students’ critical thinking development.

### 3.3 Research design

At the beginning of the course, all students completed the UFCTI to establish baseline measures of their critical thinking (CT) styles. This initial assessment served as a reference point for evaluating changes resulting from the intervention. Throughout the semester, students in the experimental group engaged in online discussion forums guided by the VaKE method, focusing on complex topics that required critical analysis and ethical deliberation. The discussions were monitored by researchers and educators, who provided guidance and support to ensure that interactions remained productive and meaningful, emphasizing the development of ethical reasoning and critical thinking.

At the end of the course, students retook the UFCTI to assess any changes in their critical thinking styles, allowing for a comparison with pre-intervention data. Additionally, students in the experimental group submitted a reflective diary, which was approximately 200–300 words in length. This diary prompted them to reflect on their experiences in the VaKE-guided forums, including questions on the benefits they gained, suggested improvements, and their understanding of cyberbullying and potential measures to address it. The inclusion of cyberbullying in the reflective diary served as an opportunity for students to engage with a relevant real-world issue, applying the critical thinking and ethical reasoning skills developed through the intervention. Table 1 visualized the differences between the experimental and control groups.

**Table 1:** Activities and instructional methods used in the experimental and control groups.

Activity	Experimental group	Control group
Pre-intervention assessment	Complete UFCTI to establish baseline CT styles	Complete UFCTI to establish baseline CT styles
Core intervention	Engage in VaKE-guided online discussion forums on complex topics requiring critical analysis and ethical deliberation	Complete individual assignments (e.g., essay-writing tasks) with no collaborative or ethical deliberation component
Discussion monitoring and support	Discussions monitored by researchers and educators, with guidance provided to promote ethical reasoning and critical thinking	No discussion monitoring or facilitator support
Post-intervention assessment	Retake UFCTI to assess changes in critical thinking styles	Retake UFCTI to assess changes in critical thinking styles
Reflective diary	Submit a reflective diary to reflect on VaKE forum experiences, including understanding of cyberbullying and ethical reasoning	No reflective diary required
Topic focus	Complex social issues such as cyberbullying, ethical reasoning, and real-world problem-solving	Focus on individual essay topics without emphasis on critical thinking or ethical deliberation

### 3.4 Data analysis

Statistical methods will be used to analyze the UFCTI responses. This will involve calculating measures of central tendency (mean, median) and variability (standard deviation) for both pre-and post-intervention assessments. Paired sample t-tests or repeated measures ANOVA will be conducted to assess the significance of any changes in critical thinking styles among students in both the control and experimental groups.

Data from the reflective diaries will be analyzed using thematic analysis (Braun and Clarke, 2006). Two coders will independently code the data to identify patterns and key themes related to student engagement, the quality of discussions, and the development of critical thinking skills. Discrepancies in coding will be resolved through discussion. The qualitative data will be analyzed using NVivo, a program designed for qualitative data analysis. The thematic findings will provide deeper insights into how the VaKE-guided forums influenced students’ critical thinking and ethical reasoning. These qualitative results will complement and enrich the quantitative findings, offering a more comprehensive understanding of the intervention’s impact.

## 4 Results

### 4.1 Results of the UFCTI

Prior to initiating the forum discussions, participants will complete a UFCTI specifically designed for this study. The 24-item *ad hoc* UFCTI (see Table 2) is structured to establish a baseline measure of the participants’ initial critical thinking capacities. The first five questions gather basic demographic information, including age, gender, major, academic year, and English College Entrance Examination scores. The remaining 19 questions employ a 5-point Likert scale to assess participants’ critical thinking approaches, anchoring to the two types of CT styles identified by Abu-Baker et al. (2021): “engager” (Q7-Q13) and “seeker” (Q6, Q14-Q24).

**Table 2:** Questions in the UFCTI.

Question		Answer
Q1	Age	Under 18/18–20/21–24/25 and over
Q2	Gender	Male/Female
Q3	Major	Arts/science/engineer
Q4	Grade	Freshman/sophomore/junior/senior
Q5	English scores of College Entrance Examination	open
Q6	I believe that most problems have more than one solution.	Likert scale 1–5
Q7	I look for opportunities to solve problems.	Likert scale 1–5
Q8	I am interested in many issues.	Likert scale 1–5
Q9	I am able to relate to a wide variety of issues.	Likert scale 1–5
Q10	I enjoy finding answers to challenging questions.	Likert scale 1–5
Q11	I am a good problem solver.	Likert scale 1–5
Q12	I am sure I can draw a reasonable conclusion.	Likert scale 1–5
Q13	I present issues in a clear and precise manner.	Likert scale 1–5
Q14	I listen carefully to the opinions of others, even when they disagree with me.	Likert scale 1–5
Q15	It’s important to be well informed.	Likert scale 1–5
Q16	I ask a lot of questions while studying.	Likert scale 1–5
Q17	I am willing to change my opinion when I am given new information I find to be credible.	Likert scale 1–5
Q18	I try to consider the facts without letting my biases affect my decisions.	Likert scale 1–5
Q19	I enjoy learning even when I am not in school.	Likert scale 1–5
Q20	I can get along with people who do not share my opinions.	Likert scale 1–5
Q21	I try to search for the truth even when it makes me feel uncomfortable.	Likert scale 1–5
Q22	I will do my best to find the right solution to the problem.	Likert scale 1–5
Q23	I am trying to seek multiple solutions to solve the problem.	Likert scale 1–5
Q24	I will ask many questions before I make a decision.	Likert scale 1–5

Likert scale 1 refers to strongly disagree; 2 stands for disagree; 3 means neutral; 4 refers to agree; 5 stands for strongly agree.

The UFCTI was administered online via Sojump, ensuring participant anonymity and allowing for voluntary and discontinuable participation. All 100 undergraduates who participated in the survey provided complete responses, resulting in a 100% valid response rate.

Table 3 showed that except for the contrastive differences in ratios of gender and major, participants of the two groups were of similar distribution in age, English scores and CT competence.

**Table 3:** Basic information of participants of the two groups.

Items			Control	Experimental	Ratio
Gender		Male	36	46	1:1.27
		Female	14	4	3.5:1
Age		Under 18	3	7	1:2.33
		18 to 20	46	42	1.10:1
		21–24	1	1	1:1
		Over 24	0	0	0
Major		Arts	11	0	0
		Science	7	8	1:1.14
		Engineer	32	42	1:1.31
English scores		Mean	109.56	110.52	1:1.01
		Maximum	120	140	1:1.17
		Minimum	90	100	1:1.11
CT competence	Pre	Mean	77.54	76.80	1.01:1
		Maximum	57	56	1.02:1
		Minimum	95	95	1:1

By means of testing the correlation of gender, age, major and CT competence scores, there shows no significant variation (GENDER: *p* = 0.438; AGE: *p* = 0.074; MAJOR: *p* = 0.091).

Tables 4, 5 indicated that the measurement of CT factors of the two groups were valid to reveal the actual competences, having standard factor loading higher than 0.5 and the value of correlation (*p* ≤ 0.05).

**Table 4:** Measurement of CT factors of the two groups.

Query	Group	CT factors	Questions	Mean	Standard error	Standard factor loading
Pre	Control	Engager	Q7	4.32	0.097	0.467
			Q8	4.04	0.128	0.815
			Q9	3.74	0.124	0.768
			Q10	3.84	0.129	0.831
			Q11	3.18	0.139	0.967
			Q12	3.88	0.123	0.761
			Q13	3.56	0.137	0.945
			Q14	4.28	0.095	0.451
		Seeker	Q6	4.58	0.081	0.330
			Q15	4.66	0.084	0.351
			Q16	3.98	0.132	0.877
			Q17	4.38	0.090	0.404
			Q18	4.36	0.094	0.439
			Q19	3.64	0.148	1.092
			Q20	4.00	0.114	0.653
			Q21	4.30	0.096	0.459
			Q22	4.22	0.100	0.502
			Q23	4.13	0.111	0.613
			Q24	4.26	0.110	0.604
	Experimental	Engager	Q7	4.30	0.108	0.582
			Q8	3.98	0.126	0.796
			Q9	3.60	0.134	0.898
			Q10	3.80	0.125	0.776
			Q11	3.40	0.118	0.694
			Q12	3.84	0.116	0.668
			Q13	3.80	0.121	0.735
			Q14	4.04	0.111	0.611
		Seeker	Q6	4.52	0.108	0.581
			Q15	4.56	0.095	0.456
			Q16	3.98	0.132	0.877
			Q17	4.38	0.090	0.404
			Q18	4.36	0.110	0.602
			Q19	3.66	0.130	0.841
			Q20	4.00	0.121	0.735
			Q21	4.30	0.096	0.459
			Q22	4.12	0.109	0.598
			Q23	4.12	0.097	0.475
			Q24	4.08	0.121	0.728
Post	Control	Engager	Q7	4.24	0.109	0.594
			Q8	3.76	0.123	0.758
			Q9	3.60	0.121	0.735
			Q10	3.64	0.102	0.521
			Q11	3.22	0.112	0.624
			Q12	3.80	0.099	0.490
			Q13	3.74	0.121	0.727
			Q14	4.04	0.099	0.488
		Seeker	Q6	4.48	0.100	0.500
			Q15	4.46	0.096	0.458
			Q16	3.62	0.117	0.689
			Q17	3.96	0.095	0.447
			Q18	4.20	0.095	0.449
			Q19	3.76	0.109	0.594
			Q20	3.80	0.107	0.571
			Q21	4.12	0.102	0.516
			Q22	4.06	0.101	0.507
			Q23	3.86	0.107	0.572
			Q24	3.90	0.108	0.582
	Experimental	Engager	Q7	4.52	0.087	0.377
			Q8	3.98	0.119	0.714
			Q9	3.62	0.106	0.567
			Q10	3.90	0.122	0.745
			Q11	3.32	0.109	0.589
			Q12	3.90	0.100	0.500
			Q13	3.78	0.112	0.624
			Q14	4.04	0.111	0.611
		Seeker	Q6	4.68	0.067	0.222
			Q15	4.74	0.069	0.237
			Q16	4.02	0.123	0.755
			Q17	4.24	0.088	0.390
			Q18	4.40	0.086	0.367
			Q19	3.74	0.139	0.972
			Q20	4.00	0.118	0.694
			Q21	4.12	0.089	0.393
			Q22	4.28	0.086	0.369
			Q23	4.04	0.121	0.733
			Q24	4.14	0.107	0.572

**Table 5:** The results of independent samples T-test of the two groups.

		Levene’s test for equality of variances					t-test to equality of means			
item		*F*	Sig.	*t*	df	Sig. (2-tailed)	Mean difference	Std error difference	95% Confidence interval of the difference	
									Lower	Upper
Query	Equal variances assumed	21.322	<0.001**	2.393	3,798	0.017*	0.065	0.027	0.012	0.119
	Equal variances not assumed				3781.782	0.017*	0.065	0.027	0.012	0.119
Style		17.177	<0.001**	−12.16	3,798	<0.001**	−0.330	0.027	−0.383	−0.277
				−11.99	3259.004	<0.001**	−0.330	0.028	−0.384	−0.276

*means that Correlation is significant at the 0.05 level (2 -tailed); ** means that Correlation is significant at the 0.01 level.

The average engagement scores for the control group decreased slightly from 3.86 in the pre-test to 3.76 in the post-test, reflecting a 0.1-point decline. In contrast, the experimental group, which participated in VaKE method-guided forums, saw a modest increase in engagement scores, rising from 3.85 in the pre-test to 3.88 in the post-test, an improvement of 0.03 points. Similarly, the average seeking scores for the control group dropped from 4.23 to 4.02, a reduction of 0.21 points, while the experimental group’s seeking scores increased marginally from 4.19 to 4.22, a gain of 0.03 points. These results suggest that the VaKE method-guided approach was effective in enhancing critical thinking, as evidenced by the improvements in both engagement and seeking scores in the experimental group.

Table 5 presents the results of the independent samples t-test conducted to compare the critical thinking (CT) styles between the control and experimental groups both before and after the intervention. The Levene’s Test for Equality of Variances indicates that for both “query” and “style” items, the assumption of equal variances was violated, as evidenced by the significant *F*-values (21.322 and 17.177, respectively, both with *p*-values less than 0.001).

The t-test for Equality of Means reveals significant differences between the two groups. For the “query” item, the t-value of 2.393 (df = 3,798, *p* = 0.017) suggests a statistically significant difference between the two groups, with a mean difference of 0.065 and a 95% confidence interval ranging from 0.012 to 0.119. Similarly, for the “style” item, the *t*-value of −12.159 (df = 3,798, *p* < 0.001) indicates a highly significant difference, with a mean difference of −0.330 and a 95% confidence interval ranging from −0.383 to −0.277.

These findings demonstrate that the experimental group, which engaged in the VaKE method-guided discussions, showed significant improvements in CT styles compared to the control group. The significant t-values and the corresponding mean differences provide quantitative evidence supporting the positive impact of the VaKE method on enhancing critical thinking in the educational context. This suggests that the VaKE approach effectively fosters a deeper engagement with critical thinking processes, as reflected in the statistically significant changes in the experimental group’s CT styles.

### 4.2 Qualitative analysis of the reflective diary

The study examined two distinct critical thinking styles: engagement and information-seeking, as categorized by Abu-Baker et al. (2021). The specific items and examples corresponding to each style are detailed in Table 6.

**Table 6:** Categories and examples of critical thinking styles.

Item	Style	Example
Opportunities to solve problems	Engagement	It is necessary to motivate discussion and communication between different groups.
Interest in many issues	Engagement	I came to know that it is not right to stand back as an onlooker.
Relate to a wide-variety of issues	Engagement	Cyberbullying does great harm on a family and the whole society. Proper measures should be taken to solve the problem, gain more knowledge of cyber bullying.
Finding answers to questions	Engagement	It’s necessary to set up laws on cyberbullying.
Good problem solver	Engagement	We should get to know cyberbullying from different angles.
Confident in conclusion	Engagement	Nothing is definite in the world. The occurrence of bullying might as well be the consequence of many factors.
Present issues clearly	Engagement	Firstly, cyberbullying frequently occurs, doing great harm to individuals. Secondly, legalization to curb cyberbully is a must for many developed countries.
Listen carefully	Seeking	We practice several steps of VaKE model and learn about different attitudes about cyberbullying.
Enjoy learning	Seeking	In the forum, I learned many skills in questioning and learn reflection.
Importance to be well-informed	Seeking	It is of great importance to communicate with other and know clearly that people vary in their way of thinking.
Ask lots of questions	Seeking	we youth can ask ourselves about what we can do for the matter, for the team, and so on.
Willing to change opinion	Seeking	Previously I reckoned the control of cyberbullying was loose, which led to many occurrences. But now I knew there were a lot of related regulations.
Consider facts without bias	Seeking	It helped me deepen my understanding of the matter and learn to take an unbiased view.
Get along with people	Seeking	I hope to increase cooperation within groups and between groups for mutual improvement.
Search for truth	Seeking	What’s more, we should search more related referential material.
Find right answers	Seeking	We need to locate the precautions and solution for cyberbullying.
Find multiple solutions	Seeking	We believe the problem can be approached from three aspects
Ask questions in decision-making	Seeking	Not found

Students participated in two forum discussions on different topics: (1) the benefits they gained from the forum and suggestions for improvement, and (2) the nature of cyberbullying and measures to address it.

Students with an engagement style tended to reflect on their personal growth and how the forum discussions influenced their perspectives, critical thinking, and understanding of social phenomena. One student commented, “The forum not only enhanced my critical thinking but also deepened my understanding of how cyberbullying affects society as a whole” (WL). Another noted, “Through these discussions, I’ve come to see the world differently, especially regarding how social issues like cyberbullying are more complex than I initially thought” (CY).

Students also highlighted the challenges they faced in the discussions, such as the need for more teacher-student interaction: “The forum lacked frequent interactions, which affected my motivation to participate” (CZL). Additionally, another student reflected, “We did not gather enough materials and did not think through the questions as thoroughly as we should have” (CJY).

Another student elaborated, “I came to know that cyberbullying is not merely attacking others online with words, but involves multifaceted actions that severely impact others’ lives, sometimes even pushing them toward suicide by spreading private information publicly on the Internet” (CY). A different participant added, “People tend to make insults, express racial prejudice, and issue threats online, which can cause significant mental and spiritual harm to the victims. This should be monitored and curbed by social media and online communities” (XKD).

A participant suggested, “We need to solve the matter by starting with relieving pressure from real-life situations, reconstructing netizens’ views on values, and legalizing prohibitions such as showing the real-time address” (WD).

Conversely, students with a seeking style were more focused on testing their ideas and demonstrating a willingness to change their views based on the discussions. For example, one student observed, “Initially, I thought cyberbullying wasn’t well-regulated, but after the forum, I realized there are many existing regulations” (CW). Another reflected, “Before the forum, I believed the loudest voices were the ones to be blamed, but now I understand that the so-called victims may not always be as innocent as they seem, thanks to the openness of the Internet” (ZJJ).

Moreover, a student emphasized the value of the VaKE method in uncovering multiple perspectives: “I now understand that we need to look at issues like cyberbullying from various angles and consider different solutions” (XKD).

A participant further noted, “We believe it is essential to regulate family education and enhance the construction of laws. Meanwhile, we need to use legal measures to protect ourselves when facing cyberbullying” (CJ).

In summary, the forum discussions provided valuable insights into how students with different critical thinking styles process information, engage with content, and adapt their perspectives. The differences between engagement and information-seeking styles were evident in the way students articulated their thoughts and reflections during and after the discussions. These reflections, captured in the students’ own words, “underscore the impact of the VaKE-guided forums in fostering both engagement and information-seeking behaviors.”

## 5 Discussion

The quantitative results from the UFCTI indicate modest but meaningful improvements in critical thinking styles for the experimental group, specifically in both engagement and information-seeking dimensions. These improvements, albeit slight, suggest that the VaKE-guided forums contributed to fostering a deeper cognitive engagement compared to the traditional essay-writing approach taken by the control group. However, the qualitative insights derived from students’ reflective diaries offer richer, context-specific explanations for these quantitative outcomes, suggesting that the VaKE method’s potential impact extends beyond mere score increases.

In the experimental group, engagement scores rose from 3.85 to 3.88, while the control group experienced a decline. The small yet positive shift in the experimental group reflects an enhanced level of interaction and active involvement in the learning process, a result that is well-supported by students’ reflections. Several participants mentioned that the forums helped them see issues like cyberbullying in new ways, illustrating the active role they played in rethinking their perspectives through dialogue and moral reasoning. This aligns with the constructivist principle of active knowledge construction, where learners are not passive recipients but active participants in shaping their understanding through discussions (Weinberger et al., 2009; Vygotsky, 1978).

One student noted that the forum “not only enhanced my critical thinking but also deepened my understanding of how cyberbullying affects society as a whole.” Such qualitative evidence reinforces the quantitative data, as it shows that the slight increase in engagement scores represents more than just statistical noise; it reflects a deeper, qualitative shift in how students approached problem-solving and ethical reflection. Moreover, these findings align with existing literature on the efficacy of moral dilemmas in educational settings. Pnevmatikos et al. (2016) and Patry (2016) emphasize that moral dilemmas stimulate cognitive disequilibrium, encouraging students to question their assumptions and reflect critically. The engagement observed in the experimental group can be understood in this light: the forums provided opportunities for students to grapple with complex, morally charged issues, pushing them to refine their ethical reasoning. This iterative process of reflection, as emphasized by Mezirow (1991)'s transformative learning theory, helps explain the observed improvements in engagement scores, as students had to continuously re-evaluate their positions.

Similarly, the experimental group showed a slight increase in information-seeking scores, rising from 4.19 to 4.22, while the control group saw a notable decline. The quantitative findings suggest that the VaKE-guided forums encouraged students to actively seek out new information to support their arguments, a behavior central to the development of critical thinking. This is further supported by qualitative data, as students frequently mentioned the need to gather more information to substantiate their views on issues such as cyberbullying. One participant reflected, “I came to know that cyberbullying is not merely attacking others online with words but involves multifaceted actions,” indicating that students were engaged in deeper inquiry to understand the complexities of the issues being discussed. This increase in information-seeking aligns with Vygotsky (1978)'s social constructivist theory, which posits that cognitive development is enhanced through social interaction and collective inquiry. The collaborative nature of the VaKE-guided forums created a learning environment in which students were not only exchanging ideas but also actively seeking out information to resolve the dilemmas presented. This collaborative element is essential for deepening students’ cognitive skills, as described by Fan et al. (2024), who argued that social interactions in collaborative learning environments drive the development of critical thinking.

Despite the improvements in both engagement and information-seeking scores, the qualitative data also reveal several challenges that may explain why the gains were modest rather than substantial. Multiple students expressed frustration over insufficient teacher-student interaction and inadequate preparation, suggesting that these factors may have limited the effectiveness of the VaKE approach. One student noted, “The forum lacked frequent interactions, which affected my motivation to participate,” while another commented, “We did not gather enough materials and did not think through the questions as thoroughly as we should have.” These insights highlight that while VaKE can be an effective tool for fostering critical thinking, its success is heavily contingent upon how it is facilitated.

The literature underscores the importance of guided inquiry and active facilitation in online learning environments. Mac Knight (2000) and Barrick and DiBenedetto (2019) both emphasize that educators play a pivotal role in guiding discussions and keeping students engaged in meaningful inquiry. The feedback from the reflective diaries aligns with these findings, suggesting that the lack of adequate facilitation may have been a barrier to more significant improvements in critical thinking outcomes. Furthermore, Vygotsky (1978)'s concept of scaffolding underscores the necessity of educator support, particularly in complex learning environments where students are engaging with challenging content.

The triangulation of quantitative and qualitative data thus offers a comprehensive picture of the VaKE method’s impact on critical thinking. The slight improvements in UFCTI scores suggest that the method can effectively enhance both engagement and information-seeking behaviors when students are actively engaged in discussion-based, moral reasoning activities. These quantitative findings are deeply enriched by the qualitative insights, which reveal that students not only participated in these activities but also experienced cognitive and ethical growth through the process of grappling with complex dilemmas. However, the challenges identified—specifically the need for more robust interaction and preparation—indicate that the effectiveness of the VaKE method is not automatic. To fully realize its potential, educators must ensure that the forums are well-facilitated, that students are adequately prepared, and that sufficient opportunities for teacher-student interaction are provided. This is particularly important in online environments, where direct interaction is more limited.

To conclude, the combination of quantitative and qualitative findings demonstrates that the VaKE method, when thoughtfully implemented and facilitated, can serve as an effective educational strategy for enhancing critical thinking among undergraduates. The slight but meaningful increases in critical thinking styles, supported by rich qualitative data, suggest that VaKE-guided forums offer a promising approach to fostering deeper cognitive and ethical engagement, also seen in the research of Pnevmatikos et al.’s (2016, 2019), Pnevmatikos et al. (2014, 2018). The students would activate and imply critical thinking skills and dispositions. However, the findings also highlight the importance of careful preparation and active facilitation in maximizing the method’s impact.

## 6 Conclusion

The study presents significant findings on the impact of the VaKE method on developing critical thinking skills among undergraduate students. Using a 24-item UFCTI to establish baseline CT styles, the responses were analyzed with SPSS 27.0, showing high reliability (Cronbach’s alpha = 0.929). The results revealed that the experimental group, which participated in VaKE-guided online forums, demonstrated modest yet meaningful improvements in both engagement and information-seeking critical thinking styles. Engagement scores increased from 3.85 to 3.88, while information-seeking scores rose from 4.19 to 4.22. In contrast, the control group, which participated in traditional essay-writing tasks, showed a decline in these dimensions, with engagement scores dropping from 3.86 to 3.76 and information-seeking scores from 4.23 to 4.02. The independent samples t-test further indicated significant differences between the experimental and control groups, suggesting that the VaKE method positively influenced critical thinking enhancement.

The qualitative insights from students’ reflective diaries offered deeper context to these quantitative findings. Participants with an engagement-oriented critical thinking style reported that the VaKE-guided forums significantly enhanced their understanding of complex social issues, such as cyberbullying, broadening their perspectives and deepening their critical thinking. These reflections indicate a progression toward more sophisticated and informed viewpoints, highlighting the cognitive and ethical development facilitated by the discussions.

However, the study also identified challenges in facilitating the forums. Students expressed the need for more teacher-student interaction and better preparation, which may have constrained the full potential of the VaKE method. These challenges underscore the importance of effective facilitation and adequate support to harness the benefits of VaKE-guided discussions fully.

Practically, the study highlights the value of VaKE-guided online forums in developing critical thinking skills in undergraduate education. Integrating moral and ethical discussions into the curriculum not only deepens cognitive engagement but also enhances students’ ability to navigate complex social issues. However, the need for adequate interaction and preparation underscores the importance of careful implementation. This research provides strong evidence that VaKE-guided forums are an effective strategy for fostering critical thinking, provided they are thoughtfully facilitated and integrated into the learning environment.

Theoretically, the study reinforces the foundation of VaKE within the constructivist learning framework. The results affirm that VaKE, by integrating moral reasoning with cognitive development, effectively promotes critical thinking, consistent with Vygotsky’s social constructivism. The observed improvements in critical thinking styles among the experimental group suggest that cognitive dispositions are dynamic and can be enhanced through structured educational interventions. Moreover, the study underscores the crucial role of educator facilitation in maximizing the effectiveness of VaKE, aligning with theories that emphasize the importance of guided inquiry and interaction in learning environments. These findings suggest that critical thinking is not merely a set of skills but a flexible cognitive style responsive to targeted educational practices.

The study provides valuable insights into the effectiveness of the VaKE method in enhancing critical thinking among undergraduates; however, it is subject to a few limitations. First, the use of convenience sampling from a single university in East China, which restricts the generalizability of the findings. Additionally, the modest improvements observed suggest that the VaKE method’s effects on critical thinking may be incremental, particularly over the short duration of the study. Conducting the research in an online environment also posed challenges, such as reduced direct interaction, potentially affecting student engagement and the effectiveness of the VaKE-guided forums. These factors suggest a need for further research with more diverse samples and contexts.

Future research should explore the long-term effects of the VaKE method on critical thinking development across diverse educational contexts and disciplines. Expanding the study to include a more varied sample from multiple institutions would enhance the generalizability of the findings. Additionally, investigating the integration of VaKE in different learning environments, such as blended or fully in-person settings, could provide deeper insights into its adaptability and effectiveness. Further studies should also examine the role of educator facilitation in greater detail, assessing how varying levels of teacher involvement impact the outcomes of VaKE-guided discussions. Finally, exploring the potential of combining VaKE with other pedagogical strategies could offer a more comprehensive approach to enhancing critical thinking in higher education.

## Data Availability

The original contributions presented in the study are included in the article, further inquiries can be directed to the corresponding author.

## References

[ref1] Abdi A. (2012). A study on the relationship of thinking styles of students and their critical thinking skills. Procedia Soc. Behav. Sci. 47, 1719–1723. doi: 10.1016/j.sbspro.2012.06.889

[ref9009] Abu-Baker N. N. AbuAlrub S. Obeidat R. F. Assmairan K. (2021). Evidence-based Practice Beliefs and Implementations: A Cross-sectional Study among Undergraduate Nursing Students. BMC Nurs 20:13. doi: 10.1186/s12912-020-00522-x 33413336 PMC7791790

[ref9010] Akins J. L. Lamm A. J. Telg R. Abrams K. Meyers C. Raulerson B. (2019). Seeking and Engaging: Case Study Integration to Enhance Critical Thinking about Agricultural Issues. Journal of Agricultural Education 60, 97–108. doi: 10.5032/jae.2019.03097

[ref3] Bailey K. Im-Bolter N. (2020). My way or your way? Perspective taking during social problem solving. J. Appl. Dev. Psychol. 66:101087. doi: 10.1016/j.appdev.2019.101087

[ref4] Barnett R. (1997). Higher education: A critical business. Buckingham: Open University Press.

[ref9006] Barrick R. K. DiBenedetto C. A. (2019). Assessing the Critical Thinking Styles of International Faculty. Journal of Advances in Education and Philosophy 3, 236–240. doi: 10.21276/jaep.2019.3.6.1

[ref9012] Braun V. Clarke V. (2006). Using Thematic Analysis in Psychology. Qualitative Research in Psychology 3, 77–101. doi: 10.1191/1478088706qp0630a

[ref5] Baviskar S. N. Hartle R. Whitney T. (2009). Essential criteria to characterize constructivist teaching: derived from a review of the literature and applied to five constructivist—teaching method articles. Int. J. Sci. Educ. 31, 541–550. doi: 10.1080/09500690701731121

[ref6] Blatt M. M. Kohlberg L. (1975). The effects of classroom moral discussion upon Children’s level of moral judgment. J. Moral Educ. 4, 129–161. doi: 10.1080/03055724750040207

[ref7] Cheong C. M. Cheung W. S. (2008). Online discussion and critical thinking skills: a case study in a Singapore secondary school. Aust. J. Educ. Technol. 24, 556–573. doi: 10.14742/ajet.1191

[ref9001] Dantas-Whitney M. (2002). Critical Reflection in the Second Language Classroom through Audiotaped Journals. System 30, 543–555. doi: 10.1016/s0346-251X(02)00046-5

[ref9] Evans J. S. B. (2008). Dual-processing accounts of reasoning, judgment, and social cognition. Annu. Rev. Psychol. 59, 255–278. doi: 10.1146/59.103006.093629, PMID: 18154502

[ref9011] Facione P. Gittens C. A. G. Facione N. C. (1995). The Disposition toward Critical Thinking. Journal of General Education 44, 1–25.

[ref10] Facione P. A. (2011). Critical thinking: what it is and why it counts. Insight Assessment 1, 1–23.

[ref12] Fan O. Y. Zhang L. Y. Wu M. Jiao P. P. (2024). Empowering collaborative knowledge construction through the implementation of a collaborative argument map tool. Internet High. Educ. 62:100946. doi: 10.1016/j.iheduc.2024.100946, PMID: 40111039

[ref13] Gay K. D. Terry B. Lamm A. J. (2015). Identifying critical thinking styles to enhance volunteer development. J. Ext. 53, 1–3. doi: 10.34068/joe.53.06.28

[ref9002] Graham P. (2005). Classroom-based Assessment: Changing Knowledge and Practice through Pre-service Teacher Education. Teaching and Teacher Education 21, 607–621.

[ref14] Gorham L. M. Lamm A. J. Rumble J. N. (2014). The critical target audience: communicating water conservation behaviors to critical thinking styles. J. Appl. Commun. 98, 42–55. doi: 10.4148/1051-0834.1092

[ref9004] Howard D. Nickels L. Coltheart M. Cole-Virtue J. (2006). Cumulative Semantic Inhibition in Picture Naming: Experimental and Computational Studies. Cognition 100, 464–482. doi: 10.1016/j.cognition.2005.02.006 16413014

[ref15] Hu J. H. (2024). The challenge of traditional teaching approach: a study on the path to improve classroom teaching effectiveness based on secondary school students’ psychology. Proceedings of the 2nd international conference on social psychology and humanity studies, 50, 213–219

[ref16] Keast S. Marangio K. (2015). Values and knowledge education (VaKE) in teacher education: benefits for science pre-service teachers when using dilemma stories. Procedia Soc. Behav. Sci. 167, 198–203. doi: 10.1016/j.sbspro.2014.12.662

[ref17] Kiran A. Khan S. A. Zia F. (2023). Measuring the effects of traditional classroom settings versus online learning environments on students’ performance and satisfaction. Int. J. Early Child. 15, 32–42. doi: 10.48047/INTJECSE/V1514.5

[ref18] Kohlberg L. (1984). The psychology of moral development: The nature and validity of moral stages. San Francisco: Harper & Row.

[ref19] Kuhn D. (2015). Thinking together and alone. Educ. Res. 44, 46–53. doi: 10.3102/0013189X15569530, PMID: 38293548

[ref20] Lamm A. J. Irani T. (2011). University of Florida critical thinking inventory (UFCTI) manual. Gainesville, FL: University of Florida.

[ref9008] Leal A. Rumble J. N. Lamm A. J. (2017). Using Critical Thinking Styles to Inform Food Safety Behavior Communication Campaigns. Journal of Applied Communications 101, 19–32. doi: 10.4148/1051-0834.1002

[ref9003] Liaw M. L. (2007). Content-based Reading and Writing for Critical Thinking Skills in an EFL Context. English Teaching & Learning 31, 45–87.

[ref9005] Lu P. Burris S. Baker M. Meyers C. Cummins G. (2021). Cultural Differences in Critical Thinking Style: A Comparison of U. S. and Chinese Undergraduate Agricultural Students. Journal of International Agricultural and Extension Education 28, 49–62. doi: 10.5191/jiaee.2021.28449

[ref21] Lu P. Oh Y. Baker M. Xu J. (2019). Assessing the dimensional validity of the university Florida critical thinking inventory (UFCTI) in Chinese: a confirmatory factor analysis. Proceedings of the 2019 Western Region AAAE Research Conference, 28, 66–72.

[ref22] Lun V. M. Fischer R. Ward C. (2010). Exploring cultural differences in critical thinking: is it about my thinking style or the language I speak? Learn. Individ. Differ. 20, 604–616. doi: 10.1016/j.lindif.2010.07.001

[ref23] Mac Knight C. (2000). Teaching critical thinking through online discussions. Educ. Q. 23, 38–41.

[ref24] Mazzocchi F. (2019). Scientific research across and beyond disciplines: challenges and opportunities of Interdisciplinartity. EMBO Rep. 20:e47682. doi: 10.15252/embr.201947682, PMID: 31040110 PMC6549017

[ref25] Mezirow J. (1991). Transformative dimensions of adult learning. San Francisco, CA: Jossey-Bass Incorporation Publishers.

[ref26] Mezirow J. (1996). Contemporary paradigms of learning. Adult Educ. Q. 46, 158–172. doi: 10.1177/074171369604600303

[ref27] Mezirow J. (2000). “Learning to think like an adult: Core concepts of transformation theory” in Learning as transformation: Critical perspectives on a theory in Progress. ed. Mezirow J. (San Francisco, CA: Jossey-Bass Incorporation Publishers), 3–34.

[ref28] Mezirow J. (2003). Transformative learning as discourse. J. Transform. Educ. 1, 58–63. doi: 10.1177/1541344603252172

[ref29] Naito T. (2013). “Moral Development” in The encyclopedia of cross-cultural psychology. ed. Keith K. D. (New York: Wiley), 891–897.

[ref30] Nisbett R. (2010). The geography of thought: How Asians and westerners think differently. New York: Simon and Schuster.

[ref31] Nussbaumer M. (2022). “Awareness of teacher roles in VaKE and capitalizing on them for teacher training” in The VaKE handbook: Theory and practice of values and knowledge education. eds. Weyringer S. Patry J. Pnevmatikos D. Børharug F. B. (Leiden: Brill Academic Publishers), 11–35.

[ref32] O'Sullivan M. Guo L. (2010). Critical thinking and Chinese international students: an east west dialogue. J. Contemp. Issues Educ. 5, 53–73. doi: 10.20355/C5NK5Z

[ref9007] Owens C. T. Lamm A. (2016). Exploring the Relationship between Critical Thinking Style and Water Conservation Behavior: Implications for Extension. Journal of Agricultural Education 57, 119–130. doi: 10.5032/jae.2016.04119

[ref33] Park E. L. Choi B. K. (2014). Transformation of classroom spaces: traditional versus active learning classroom in colleges. High. Educ. 68, 749–771. doi: 10.1007/s10734-014-974-0

[ref34] Paton M. (2005). “Is critical analysis foreign to Chinese students?” in Communication skills in university education: The international dimension. eds. Manalo E. Wong-Toi G. (Aukland: Pearson Education New Zealand), 1–11.

[ref35] Patry J.-L. (2014). “Die Viabilität und der Viabilitäts-Check von Antworten” in Fragen! Antworten? eds. Giordano C. Patry J.-L. (Freiburger Sozialanthropologische Studien. Vienna, Austria: Lit), 11–35.

[ref36] Patry J.-L. (2016). “Inquiry learning arrangements from the perspective of critical Multiplism and related concepts” in Theory of inquiry learning arrangements: Research, reflection, and implementation. eds. Reitinger J. Haberfellner C. Brewster E. Kramer M. (Kassel: Kassel University Press), 171–186.

[ref37] Patry J.-L. Weinberger A. Weyringer S. Nussbaumer M. (2013). “Combining values and knowledge education” in The handbook of educational theories. eds. Irby B. J. Brown G. Lara-Alecio R. Jackson S. (Charlotte, NC: Information Age Publishing), 565–579.

[ref9013] Pnevmatikos D. Christodoulou P. Fachantidis N. (2018). Promoting Critical Thinking Dispositions in Children and Adolescents Through Human-robot Interaction with Socially Assistive Robots. TCH-EDU. doi: 10.1007/978-3-030-20954-4_11

[ref9014] Pnevmatikos D. Christodoulou P. Georgiadou T. Lithoxoidou A. (2014). Undergraduate Students’ Conceptualization of Critical Thinking and Their Ideas for Critical Thinking Acquisition. Education Sciences 13:416. doi: 10.3390/edusci13040416

[ref39] Pnevmatikos D. Christodoulou P. Georgiado T. (2019). Promoting critical thinking in higher education through the values and knowledge education (VaKE) method. Stud. High. Educ. 44, 892–901. doi: 10.1080/03075079.2019.1586340

[ref40] Pnevmatikos D. Christodoulou P. (2018). “Promoting conceptual change through values and knowledge education (VaKE)” in Professionals’ ethos and education for responsibility. eds. Weinberger A. Biedermann H. Patry J.-L. Weyringer S. (Leiden, The Netherlands: Brill), 63–74.

[ref41] Pnevmatikos D. Patry J.-L. (2014). “Combining values and knowledge teaching through the Dilemma's discussion” in Instruction and “building” issues in multicultural school environment. eds. Katsarou E. Liakopoulou M. (Thessaloniki, Greece: Ministry of Education), 555–575.

[ref42] Pnevmatikos D. Patry J.-L. Weinberger A. Linortner L. Weyringer S. Maron R.. (2016). “Combining values and knowledge education for lifelong transformative learning” in Lifelong learning: Concepts, benefits, and challenges. eds. Panitsides E. Talbot J. (New York: Nova Science), 109–134.

[ref43] Rudd R. Baker M. Hoover T. (2000). Undergraduate agriculture student learning styles and critical thinking abilities: is there a relationship? J. Agric. Educ. 41, 2–12. doi: 10.5032/jae.2000.03002

[ref44] Saprudin S. Liliasari S. Prihatmanto A. S. Setiawan A. (2019). Pre-service physics teachers’ thinking styles and its relationship with critical thinking skills on learning interference and diffraction. J. Phys. Conf. Ser. 1157:032029. doi: 10.1088/1742-6596/1157/3/032029

[ref45] Sternberg R. J. Waggner R. K. (1992). Thinking styles inventory. New Haven: Yale University.

[ref46] Tan C. (2017). Teaching critical thinking: cultural challenges and strategies in Singapore. Br. Educ. Res. J. 43, 988–1002. doi: 10.1002/BERJ.3295

[ref47] Vygotsky L. S. (1978). Mind in society: The development of higher psychological processes. Cambridge: Harvard University Press.

[ref48] Weinberger A. (2006). “Kombination von Werterziehung und Wissenserwerb. Evaluation des konstruktivistischen Unterrichtsmodells VaKE (Values and Knowledge Education)” in In der Sekundarstufe I [combination of values education and knowledge construction. Evaluation of the learning method VaKE] (Hamburg, Germany: Dr. Kovač).

[ref49] Weinberger A. (2015). A moral case analysis approach to promote the professional ethos of teachers. Paper presented at the 16th European conference for research on learning and instruction, Limassol.

[ref50] Weinberger A. Nussbaumer M. (2019). “VaKE+: fostering learning performance in cognitive heterogeneous classes in lower secondary schools” in The VaKE handbook: Theory and practice of values and knowledge education. eds. Weyringer S. Patry J. Pnevmatikos D. Børharug F. B. (Leiden: Brill Academic Publishers), 36–55.

[ref52] Weinberger A. Patry J.-L. Weyringer S. (2009). “Wertewildnis und Wissenserwerb im Fachunterricht–Gelingensbedingungen für VaKE-Methoden” in Auf der Suche nach den Werten. Ansätze und Modelle zur Wertereflexion in der Schule. eds. Seyfried C. Weinberger A. (Lit: Vienna, Austria), 181–210.

[ref55] Yeh M. Chen H. (2003). Comparison of affective dispositions toward critical thinking across Chinese and American baccalaureate nursing students. J. Nurs. Res. 11, 39–45. 12695978 10.1097/01.jnr.0000347617.29413.96

[ref56] Zhang L. Sternberg R. J. (2001). “Thinking styles across cultures: their relationships with student learning” in Perspectives on thinking, learning, and cognitive styles. eds. Sternberg R. J. Zhang L. (Mahwah, NJ: Lawrence Erlbaum Associates Incorporation), 197–226.

